# The causative drugs and the prevalence of severe bullous hypersensitivity reactions

**DOI:** 10.1186/2045-7022-4-S3-P16

**Published:** 2014-07-18

**Authors:** Ekaterina Vertieva, Pavel Kolkhir, Natalia Teplyuk

**Affiliations:** 1 I.M. Sechenov First Moscow State Medical University, Russia; 2 I.M. Sechenov First Moscow State Medical University, Allergy Department, Russia; 3 I.M. Sechenov First Moscow State Medical University, Dermatology Department, Russia

## Background

According to recent studies about 1-3% hypersensitivity reactions are accompanied by skin eruptions. Among these toxic epidermal necrolysis (TEN) and Stevens-Johnson syndrome (SJS) are rare and the most severe adverse cutaneous drug reactions that predominantly involve the skin and mucous membranes. TEN and SJS affecting approximately 1-4/1,000,000 annually with average reported mortality rate 25-35% (TEN), and 1-5% (SJS). The aim of this work was to study the frequency of severe bullous delayed allergic reactions, like TEN and SJS, their clinical presentations, causative drugs, treatment and the diagnostic approach required.

## Method

We followed up 33 patients (7 males, 26 females) aged 21-80 years with various severe bullous dermatoses in dermatology department of our hospital.

## Results

Most of the patients had drug hypersensitivity reactions (n=22) and most of them were female older than 70 years. 3 of them had TEN and 7 suffered from SJS. The causative drugs of TEN and SJS were sulfonamides (23%, n=5), NSAIDs (14%, n=3) and anticonvulsants (9%, n=2). The most common drug was sulfamethoxazole (18%). The most severe reactions were caused by sulfamethoxazole (13%), metamizole (4%) and carbamazepine (9%). Mortality rate was about 4% (n=1).

## Conclusion

The results of our study indicate that TEN and SJS may occur in about 32% patients with severe bullous dermatoses. Sulfanonamides, NSAIDs and anticonvulsants are the most frequently implicated drugs. Diagnosis relies mainly on clinical symptoms together with the results of a skin biopsy. Due to the high risk of mortality, management of patients with severe bullous dermatoses, especially TEN and SJS, requires rapid identification and interruption of the culprit drug, specialized supportive care and immunomodulatory therapy.

